# PKA activation and endothelial claudin-5 breakdown in the schizophrenic prefrontal cortex

**DOI:** 10.18632/oncotarget.21850

**Published:** 2017-10-16

**Authors:** Keisuke Nishiura, Naoki Ichikawa-Tomikawa, Kotaro Sugimoto, Yasuto Kunii, Korehito Kashiwagi, Mizuko Tanaka, Yuichi Yokoyama, Mizuki Hino, Takashi Sugino, Hirooki Yabe, Hitoshi Takahashi, Akiyoshi Kakita, Tetsuya Imura, Hideki Chiba

**Affiliations:** ^1^ Department of Basic Pathology, Fukushima Medical University School of Medicine, Fukushima, Japan; ^2^ Department of Neuropsychiatry, Fukushima Medical University School of Medicine, Fukushima, Japan; ^3^ Department of Psychiatry, Aizu Medical Center, Fukushima Medical University, Fukushima, Japan; ^4^ Department of Pathology, Brain Research Institute, Niigata University, Niigata, Japan; ^5^ Department of Diagnostic Pathology, Shizuoka Cancer Center, Shizuoka, Japan; ^6^ Department of Surgical Pathology, Kyoto Prefectural University of Medicine, Kyoto, Japan

**Keywords:** blood-brain barrier, tight junction, claudin, protein kinase A, schizophrenia, Pathology Section

## Abstract

Schizophrenia is thought to be caused by a combination of genetic and environmental factors; however, its pathogenesis remains largely unknown. Here, we focus on the endothelial tight-junction protein claudin-5 (CLDN5), because the *CLDN5* gene is mapped to the schizophrenia-associated 22q11.2 deletion region, and a single nucleotide polymorphism in the *CLDN5* locus is also linked to schizophrenia. We show, by RT-qPCR and immunohistochemistry, that the expressions of CLDN5 mRNA and protein are significantly increased and decreased, respectively, in the schizophrenic prefrontal cortex (PFC) compared with control PFC. These changes were not observed in the schizophrenic visual cortex (VC), and neither the density nor diameter of the CD34-positive microvessels was altered in the schizophrenic PFC or VC. Interestingly, protein kinase A (PKA) was activated in the microvascular and perivascular regions of the schizophrenic PFC, and the pPKA-positive microvascular endothelial cells occasionally exhibited focal loss of CLND5. Since we previously demonstrated that cAMP induced *CLDN5* mRNA expression and size-selective loosening of the endothelial barrier in PKA-independent and -dependent manners, respectively, a similar mechanism could contribute to the discrepancy between mRNA and protein expression of CLDN5 in the schizophrenic PFC. Taken collectively, these findings provide novel insights into the pathophysiology of schizophrenia.

## INTRODUCTION

Schizophrenia is a complex psychotic disorder with an estimated prevalence of nearly 1% [1]. Among cortical areas that are functionally and/or structurally affected in schizophrenia, the dorsolateral prefrontal cortex (PFC) is most commonly involved, and its volume is markedly decreased in the chronic state [2, 3]. One of the strongest established genetic risk factors is the 22q11.2 microdeletions that cause 22q11.2 deletion syndrome (also known as velocardiofacial syndrome or DiGeorge syndrome) [4-6]. Indeed, the risk of developing schizophrenia rises 20-fold in individuals with 22q11.2 microdeletions [7], strongly suggesting that the region overlapping 90 genes contains one or more susceptibility genes for schizophrenia [8, 9]. Although genetic and environmental factors increase the risk of schizophrenia [10-12], the pathophysiology remains poorly understood.

The blood-brain barrier (BBB) highly limits the movement of molecules, ions, and cells between the blood circulation and the central nervous system to protect the brain. Brain microvascular endothelial cells (BMVECs) possessing well-developed tight junctions are primarily critical for the BBB, though the surrounding components, such as pericytes, astrocytic endfeet, and basement membranes that consist of various extracellular matrix proteins, also contribute to the establishment of the BBB [13-16]. Breakdown of the BBB has been reported in various neurological disorders, including brain ischemic stroke, edema, infections, epilepsy, multiple sclerosis, Alzheimer’s disease, and Parkinson’s disease [14, 15, 17, 18].

An association between the BBB dysfunction and schizophrenia has also been proposed [19, 20]. Interestingly, the *Claudin-5 (CLDN5)* gene, whose product represents the major component of barrier-forming tight junctions in endothelial cells [21, 22], is mapped to the 22q11.2 deletion region [8, 9]. In fact, CLDN5 was originally identified as a transmembrane protein deleted in velocardiofacial syndrome (TMVCF) [23]. A single nucleotide polymorphism located in the 3’-untranslated region of the *CLDN5* locus is also related to schizophrenia [24, 25].

CLDNs are capable of forming tight-junction strands [26], and thus the backbone of the tight junctions. Among the members of the CLDN family, CLDN5 is indispensable for the BBB. CLDN5-deficient mice exhibit a size-selective loosening of the BBB against small molecules (less than 800 Da), and die within 10 h after birth [22]. We previously demonstrated that cAMP activated gene expression of CLDN5 in porcine BMVECs in a protein kinase A (PKA)-independent manner [27]. We also uncovered that a phosphorylation site of human and mouse CLDN5 for PKA, Thr207 (RRPT) in the cytoplasmic terminal domain, was responsible for a size-selective loosening of the endothelial barrier against small molecules [28].

Therefore, in the present study, we compared *CLDN5* mRNA levels and CLDN5- or pPKA-positive fields as well as the diameter and density of BMVECs in both the PFC and visual cortex (VC) between schizophrenic and control specimens. Our results highlight that immuno-reactive signals for CLDN5 and pPKA are prominently diminished and induced, respectively, in the microvascular regions of the schizophrenic PFC.

## RESULTS

### Expression of *CLDN5* mRNA is increased in the schizophrenic PFC

**Figure 1:**
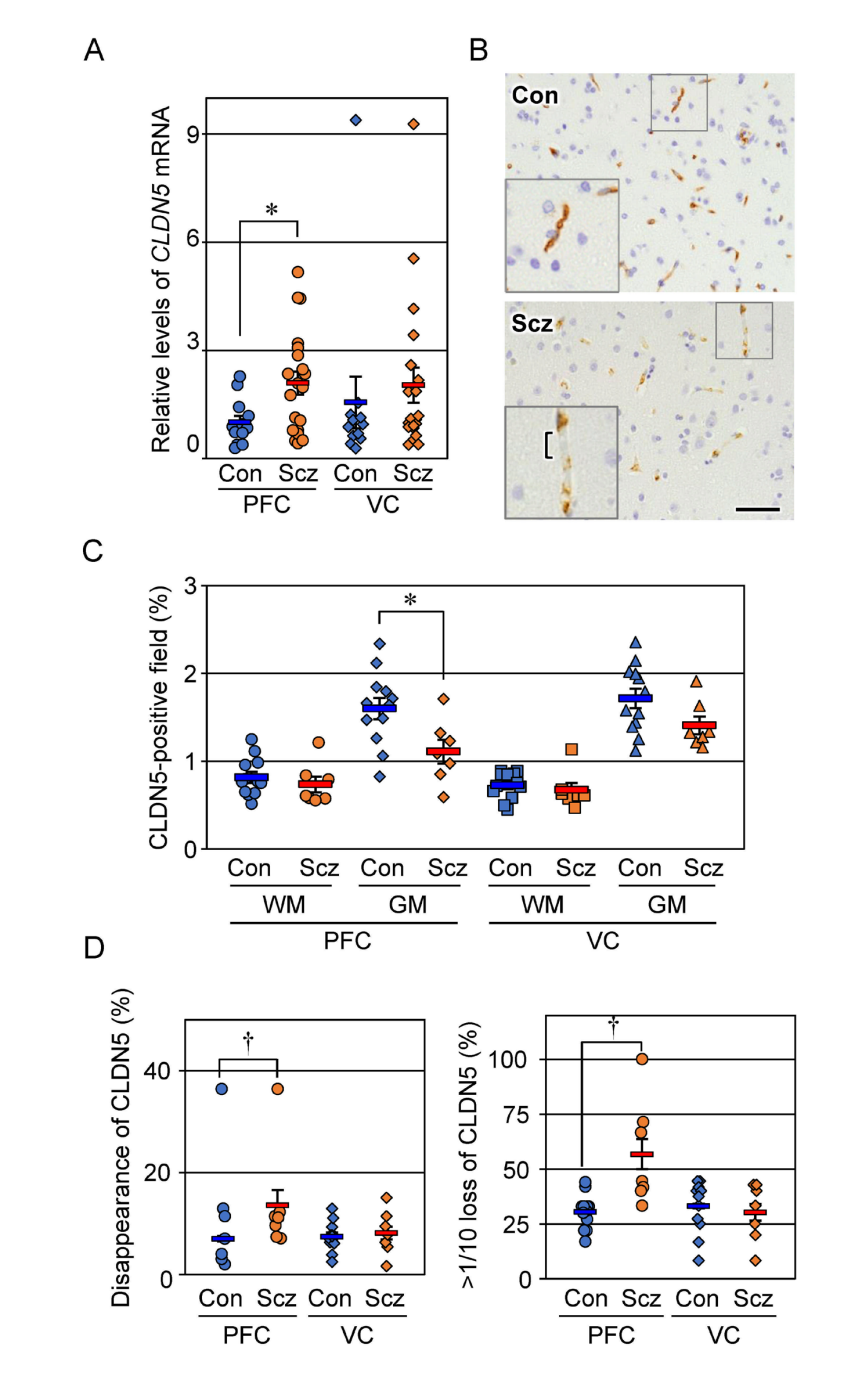
Expression of CLDN5 mRNA and protein is reciprocally modulated in the PFC gray matter of schizophrenia (**A**) The mRNA levels of *CLDN5* in the indicated brain tissues were determined by real-time PCR, and normalized to the corresponding *GAPDH* levels. The values are plotted relative to the mean value in the control PFC, which was taken as 1, and represent the mean ± SEM (Con; n=12, Scz; n=20). Con; control, Scz; schizophrenia, PFC; prefrontal cortex, VC; visual cortex. ^*^P < 0.05. (**B**) The PFC gray matter tissues of Con and Scz subjects were subjected to immunostaining with the anti-CLDN5 antibody. Bracket indicates the discontinuous CLDN5-immunoreactivity. Scale bar, 50 µm. (**C**) The percentages of CLDN5-positive field are plotted, and the mean ± SEM (Con; n=12, Scz; n=7) are represented. GM; gray matter, WM; white matter. ^*^P < 0.05. (**D**) The percentages of both CLDN5 disappearance and over 1/10 loss of CLDN5 signal in the vessel length axis are plotted, and the mean ± SEM (Con; n=12, Scz; n=7) are represented. ^†^*P* < 0.01.

We first determined by RT-qPCR the expression levels of *CLDN5* mRNA in control and schizophrenic brain tissues (Figure 1A). *CLDN5* mRNA levels in the schizophrenic PFC samples were significantly increased compared with those in the control PFC specimens. On the other hand, there were no obvious differences in the expression levels of *CLDN5* transcripts between the schizophrenic and control VC. Thus, expression of *CLDN5* mRNA was enhanced in a brain region-specific manner of schizophrenia.

### CLDN5-immunoreactive area is decreased in the schizophrenic PFC gray matter

We next evaluated by immunohistochemistry the expression of CLDN5 protein in control and schizophrenic cortical regions. CLDN5 was observed in the cortical microvessels of both the control and schizophrenic brain tissues, showing that the endothelial-specific distribution is maintained in schizophrenia. Interestingly, however, CLDN5-positive signals in the PFC gray matter of the schizophrenic group were decreased compared with those of the controls, and occasionally exhibited discontinuous staining patterns (Figure 1B). Such changes were not apparent in the PFC white matter, nor the VC white or gray matter. We therefore quantified the CLDN5-immunoreactive area in each cortical region of the control and schizophrenic groups. As shown in Figure 1C, the CLDN5-positive field in the PFC gray matter of schizophrenic subjects was significantly diminished compared with that of the controls. By contrast, no significant differences were detected in other brain regions examined between both groups. In addition, the proportion of CLDN5 disappearance in the vessel length axis of the PFC gray matter of schizophrenic subjects was significantly increased compared with that of the controls (Figure 1D). The CLDN5-immunopositive fraction in the gray matter was higher than that in the white matter in both groups, which is likely to reflect differences in microvessel density between the gray and white matter, as shown below.

### Microvessel density and diameter are not altered in schizophrenia

**Figure 2:**
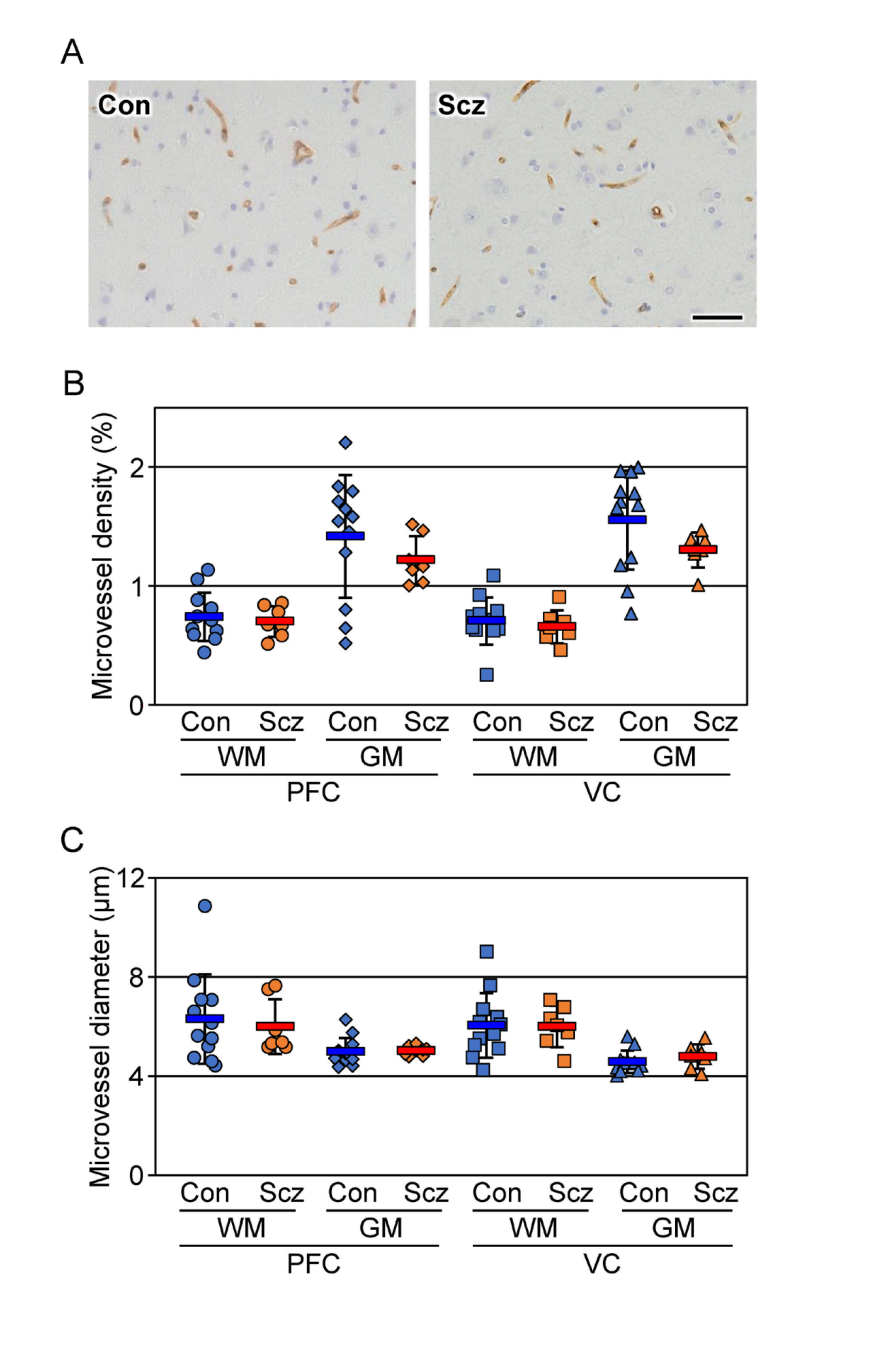
No changes are detected in the microvessel density and diameter of the schizophrenic brain cortex (**A**) The PFC gray matter tissues of the control (Con) and schizophrenia (Scz) groups were subjected to immunostaining with the anti-CD34 antibody. Scale bar, 50 µm. The percentages (**B**) and diameter (**C**) of the CD34-positive area are plotted, and the mean ± SEM (Con; n=12, Scz; n=7) are represented. PFC; prefrontal cortex, VC; visual cortex, GM; gray matter, WM; white matter. ^*^P < 0.05.

To exclude the possibility that the decreased CLDN5-positive field in the schizophrenic PFC gray matter could be due to a lower density in its microvessels, serial sections were immunostained with the endothelial marker CD34, and both the density and diameter of the microvessels were quantified (Figure 2A-C). No significant differences were detected in the microvessel density or diameter of each brain region examined between the control and schizophrenic groups as reported previously [29, 30], strongly suggesting that the expression of CLDN5 protein was selectively reduced in the schizophrenic PFC gray matter. The microvessel densities in the gray matter were higher than those in the white matter in both groups, which most likely corresponds to a well-known physiological difference between the two brain regions.

### PKA is activated in the schizophrenic PFC

**Figure 3:**
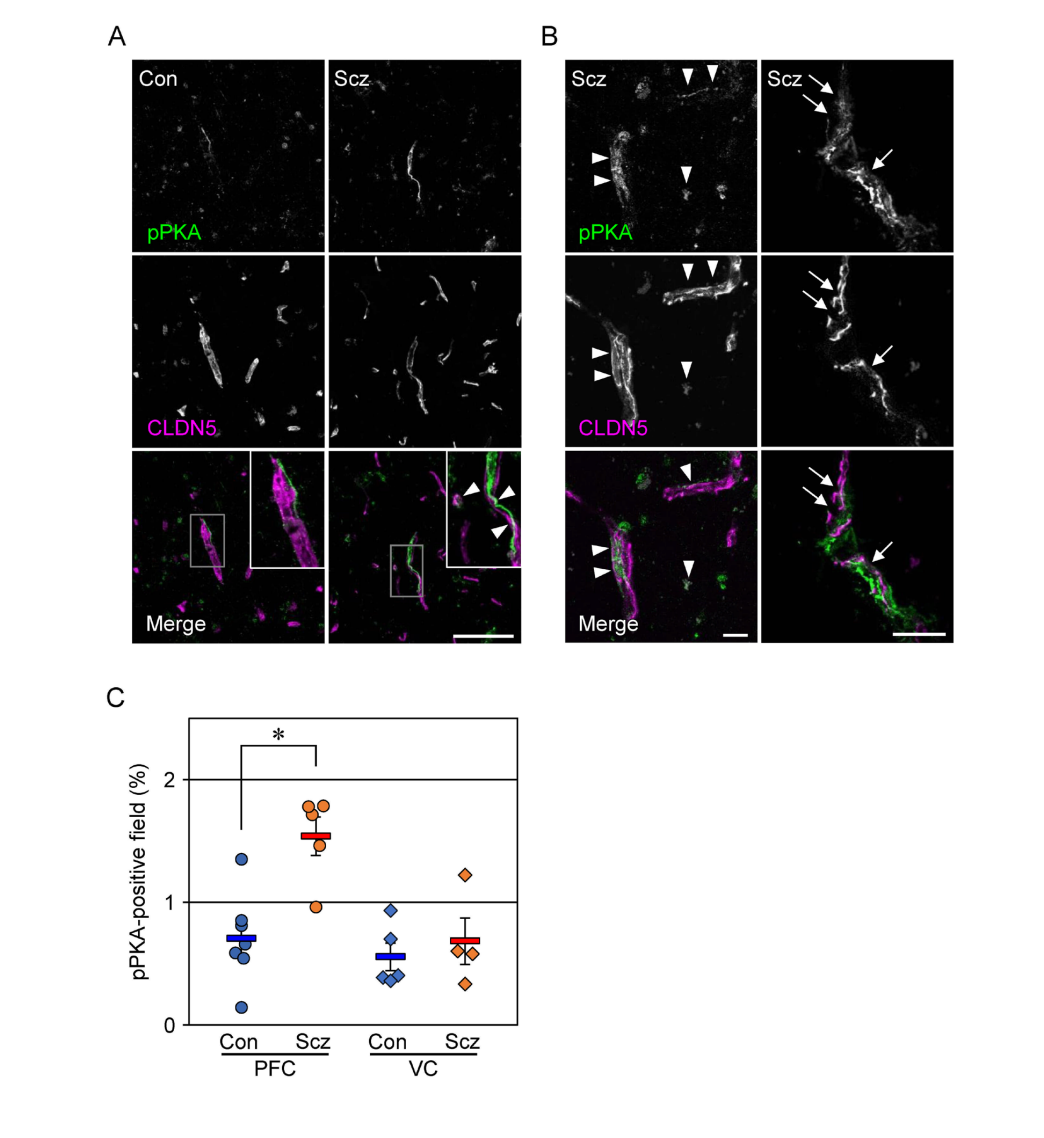
PKA is activated in the PFC of schizophrenia (**A**, **B**) Confocal images of the control (Con) and schizophrenic (Scz) PFC gray matter tissues stained for pPKA with CLDN5. Arrowheads indicate colocalization of pPKA and CLDN5, and arrows show the discontinuous CLDN5-immunoreactivity in pPKA-positive microvessels. Scale bars, (A) 100 µm; (B) 20 µm. (**C**) The percentages of the pPKA-positive field are plotted, and the mean ± SEM (Con/PFC; n=7, Scz/PFC; n=5, Con/VC; n=5, Scz/VC; n=4) are represented. PFC; prefrontal cortex, VC; visual cortex, GM; gray matter, WM; white matter. ^*^P < 0.01.

We previously showed that cAMP activated PKA to phosphorylate Cldn5 protein in cultured porcine and rat endothelial cells, resulting in size-selective loosening of the endothelial barrier, and that it simultaneously elevated the levels of Cldn5 mRNA in a PKA-independent manner [27, 28]. Therefore, we hypothesized that the discrepancy between mRNA and protein expression of CLDN5 in the schizophrenic PFC could be caused by similar molecular mechanisms. To verify whether PKA is activated in the PFC microvessels of schizophrenc subjects, frozen sections were immunostained for the phosphorylated catalytic subunit of PKA with CLDN5. As shown in Figure 3A, the pPKA-immunoreactivity in the PFC of schizophrenic subjects was enhanced compared with those of the controls. In addition, the pPKA signals were often distributed close to CLDN5-positive BMVECs, and were at least in part colocalized with CLDN5 (Figure 3A, B). Intriguingly, the pPKA-positive microvessels occasionally exhibited irregular discontinuity of CLDN5-immunoreactivity in the schizophrenic PFC (Figure 3B, right). Moreover, quantitative analysis demonstrated that the pPKA-positive field in the schizophrenic PFC was significantly higher than that in the control PFC, and that no differences were observed regarding VC between the two groups (Figure 3C). Thus, PKA appeared to be selectively activated in BMVECs and the surrounding cells of the schizophrenic PFC, in which CLDN5 breakdown was detected.

### Endothelial barrier against fibrinogen and IgG is maintained in the schizophrenic PFC

**Figure 4:**
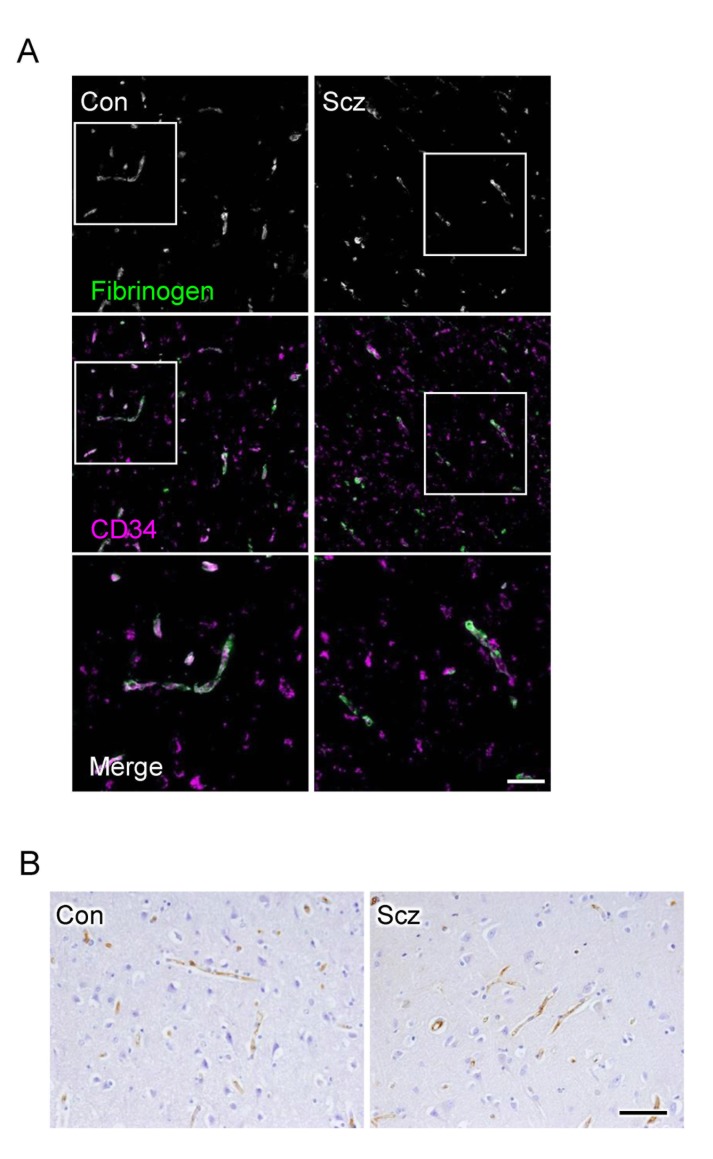
No leakage of fibrinogen and IgG are observed from microvessels in the schizophrenic PFC (**A**) Confocal images of the control (Con) and schizophrenic (Scz) PFC gray matter tissues stained for fibrinogen with CD34. (**B**) Images of Con and PFC gray matter tissues immunostained for IgG. Scale bars, (**A**) 100 µm; (**B**) 50 µm.

Since CLDN5 contributes to the endothelial barrier against small molecules (less than 800 Da) but not against larger ones [22], we subsequently checked the extravasation of fibrinogen (340 kDa) and IgG (160 kDa) in the PFCs of the schizophrenic and control groups. As suspected, the leakage of these serum proteins from blood vessels was not observed in both groups (Figure 4A, B), indicating that the barrier against large molecules is maintained in the schizophrenic PFC.

## DISCUSSION

CLDN5 is absolutely required for the development and maintenance of the BBB, since the barrier against small molecules (less than 800 Da) is severely disrupted in CLDN5-null mice [22]. Focal loss of CLDN5 is also associated with the BBB dysfunction in mice [31]. Moreover, a close relationship between the diminished CLDN5 expression in BMVECs and the BBB breakdown has been reported in a range of human neurological disorders [14, 15, 17, 18]. Thus, CLDN5 could represent for the tightness of the BBB. In the present study, we found by quantitative immunohistochemical analysis that expression of the CLDN5 protein in microvessels of the schizophrenic PFC gray matter was significantly decreased compared with that in control subjects. By contrast, no differences in CLDN5 protein expression were detected in the VC white or gray matter between the schizophrenic and control groups. Although we cannot evaluate the leakage of small serum proteins or peptides due to the absence of such endogenous markers, the endothelial barrier against fibrinogen and IgG was well maintained in the schizophrenic PFC, suggesting a lack of the massive BBB breakdown. Taken together, our results indicate that a significant reduction in the expression of CLDN5 protein occurs selectively in the PFC gray matter in patients with schizophrenia, most probably resulting in focal BBB breakdown for small molecules. Furthermore, brain region-selective BBB dysfunction may be also involved in the pathogenesis of other neurological disorders.

Interestingly, microvascular and perivascular PKA activation appeared to be observed in the schizophrenic PFC but neither in the schizophrenic VC nor in the control PFC or VC. More importantly, the pPKA-positive BMVECs in the schizophrenic PFC occasionally displayed focal loss of CLDN5. In other words, the activity of PKA and amount of CLDN5 protein were conversely regulated in the microvessels. In this respect, it should be noted that we previously identified a phosphorylation site for PKA at Thr207 in the cytoplasmic domain of CLDN5 [27]. We next generated rat lung endothelial cells expressing doxycycline-inducible wild-type CLDN5 or the CLDN5 mutant with a substitution of Thr207 for an Ala residue, and demonstrated that phosphorylation of this site by PKA caused size-selective loosening of the endothelial barrier [28]. The significance of the phosphorylation of CLDN5 at Thr207 in the BBB breakdown has also been reported in human immunodeficiency virus-1 encephalitis [32]. Thus, we conclude that the enhanced PKA activity could lead to the breakdown of CLDN5 in the BMVECs of the schizophrenic PFC.

Another issue that should be discussed is the discrepancy between mRNA and protein expression of CLDN5 in the schizophrenic PFC. Intriguingly, we previously demonstrated that cAMP induced mRNA expression and Thr207-phosphorylation of CLDN5 in BMVECs in PKA-independent and -dependent fashions, respectively [27]. Since the cAMP signalling [33, 34] and downstream PKA signalling reported in the present study are aberrantly activated in the schizophrenic PFC, this cascade could be responsible for the induced expression of *CLDN5* mRNA and the breakdown of CLDN5 protein. Hence, the significant up-regulation of *CLDN5* mRNA is detected in the schizophrenic PFC but, nevertheless, the phosphorylation and subsequent degradation of CLDN5 seems to predominate in the induced gene expression.

In conclusion, we found that PKA activation and CLDN5 breakdown occurred in the microvessels of schizophrenic PFC. The molecular mechanism underlying the PKA stimulation is unknown. However, since many different members of G protein-coupled receptors and their corresponding ligands are known to both positively and negatively regulate the cAMP/PKA pathway [35, 36], they may contribute to PKA activation in the microvessels of the schizophrenic PFC. Our findings provide new insights into the pathophysiology of schizophrenia.

## MATERIALS AND METHODS

### Cases and brain tissues

Postmortem human brain tissues were obtained from the Fukushima Postmortem Brain and DNA Bank for Psychiatric Research (Fukushima PMB/DNA Bank) and the Niigata University Brain Research Institute. All schizophrenic subjects had been diagnosed according to the DSM-IV (Diagnostic and Statistical Manual of Mental Disorders, Fourth Edition) criteria, and informed consent was obtained from the next of kin for each subject. The schizophrenia and control subjects had no histories of other neurological disorders, alcoholism, or drug abuse, and were matched for sex, age, and postmortem interval as much as possible (Supplementary Table 1). Brains were collected and cut coronally in 10-μm slices, from which the PFC (Brodmann area [BA] 10) and the VC (BA17) were dissected. The samples were either stored at −80°C until use or fixed with formalin and embedded in paraffin blocks.

All experimental protocols were approved by the Ethical Committee of Fukushima Medical University and carried out in accordance with relevant guidelines and regulations.

### Real-time PCR

Total RNA was isolated using TRIzol reagent (Invitrogen, New York, NY), and complementary DNAs were produced from 1 µg of DNase-treated RNA using a random primer according to the manufacturer’s instructions (Takara Bio, Kusatsu, Japan). Target genes were quantified by real-time PCR (StepOne; Applied Biosystems, Foster City, CA) using SYBR Green (Fast SYBR Green PCR Master Mix; Applied Biosystems). The primers used are shown below. All measurements were performed in triplicate, and the expression levels of *CLDN5* gene were normalized using the expression of the housekeeping gene *GAPDH*. The relative amount of transcript was estimated by the standard curve method.

*CLDN5*: product size 302 bp, Refseq; NM_001130861.1

Forward; 5’-CTGTTTCCATAGGCAGAGCG-3’

Reverse; 5’-AAGCAGATTCTTAGCCTTCC-3’

*GAPDH*: product size 117 bp, Refseq; NM_002046.5

Forward; 5’-TTGTTGCCATCAATGACCCC-3’

Reverse; 5’-TGACAAGCTTCCCGTTCTCA-3’

### Immunohistochemistry

For bright-field immunohistochemistry, tissues were embedded in paraffin and sectioned at a thickness of 4 μm with a microtome. Antigen retrieval was subsequently performed by microwaving in 10 mM sodium citrate buffer (pH 6.0) or by incubation with 0.5% trypsin. After blocking using 5% skimmed milk (Morinaga Milk Industry, Tokyo, Japan), the sections were incubated overnight at 4°C with primary antibodies, rabbit anti-CLDN5 (1:200, IBL, Fujioka, Japan; [27]), mouse anti-CD34 (1:100, Nichirei, Tokyo, Japan), and rabbit anti-IgG (1:1000, Abcam, Cambridge, MA, USA). After washing with PBS, secondary antibody reaction was performed by using Histofine Simple Stain MAX-PO (MULTI) Kit (Nichirei) with DAB as a chromogen according to the manufacturer’s instructions.

For immunofluorescence staining, sections were prepared at a thickness of 20 μm from snap-frozen tissues, and fixed in 100% methanol for 10 min at −20^o^C. After blocking with 5% donkey serum and 2% bovine serum albumin in either TBS or PBS for 30 min, they were incubated for 1 h at room temperature with rabbit anti-phosphorylated PKA (pPKA) (1:500, Santa Cruz Biotechnology, Dallas, TX, USA), mouse anti-CLDN5 (1:200, Invitrogen) and rabbit anti-fibrinogen (1:200, Dako, Glostrup, Denmark). They were subsequently reacted with either Alexa fluor 488 donkey anti-rabbit IgG or Cy3 donkey anti-mouse IgG (Thermo Fisher Scientific, Waltham, MA, USA).

All samples were examined and photographed using bright-field microscopy (OLYMPUS BX61, Olympus, Tokyo, Japan) and scanning confocal laser microscopy (FV1000; Olympus). Ten stacks of 0.5-μm-thick optical slices were collected through the z axis of tissue sections of regions to be analyzed.

### Quantitative morphometric analysis

For quantification of immunoreactive signals for CLDN5 and CD34, five microscopic fields (magnification ×200, 440×330 µm^2^) per region were randomly selected, and immunoreactive area fraction was determined using ImageJ software (ver 1.49). Briefly, DAB-stained images were binarized and the percentage of immunostained area per total area was calculated. The CLDN5-positive vessels with a long axis over 100 pixels (80 µm) were also picked out, and the percentage of CLDN5 disappearance was quantified. In addition, blood vessel diameter was also measured (more than 120 vessels from each region) using CD34-stained sections.

For quantification of pPKA signals, five fields (magnification ×200, 635×635 µm^2^) per region were randomly chosen, and the immunoreactive area was calculated using ImageJ software.

### Statistical analysis

The statistical significance of differences between the control and schizophrenic groups in each region was evaluated by the Mann-Whitney U tests for the non-parametric analysis, and analyzed by SPSS software (ver. 21, IBM, Armonk, NY).

## SUPPLEMENTARY MATERIALS TABLE


